# *HMGA1* and *HMGA2* expression and comparative analyses of *HMGA2*, *Lin28* and let-7 miRNAs in oral squamous cell carcinoma

**DOI:** 10.1186/1471-2407-14-694

**Published:** 2014-09-23

**Authors:** Katharina Anna Sterenczak, Andre Eckardt, Andreas Kampmann, Saskia Willenbrock, Nina Eberle, Florian Länger, Sven Kleinschmidt, Marion Hewicker-Trautwein, Hans Kreipe, Ingo Nolte, Hugo Murua Escobar, Nils Claudius Gellrich

**Affiliations:** Small Animal Clinic, University of Veterinary Medicine Hannover, Bünteweg 9, 30559 Hannover, Germany; Department of Oral and Maxillofacial Surgery, Hannover Medical School, Carl-Neuberg-Strasse 1, 30625 Hannover, Germany; Institute for Pathology, Hannover Medical School, Carl-Neuberg-Strasse 1, 30625 Hannover, Germany; Department of Pathology, University of Veterinary Medicine Hannover, Bünteweg 17, 30559 Hannover, Germany; Division of Medicine, Department of Haematology/Oncology, University of Rostock, Ernst-Heydemann-Strasse 6, 18057 Rostock, Germany

**Keywords:** Squamous cell carcinoma, HMGA1, HMGA2, let-7, mir-98, Lin28, Animal model, Dogs, Comparative oncology

## Abstract

**Background:**

Humans and dogs are affected by squamous cell carcinomas of the oral cavity (OSCC) in a considerably high frequency. The high mobility group A2 (HMGA2) protein was found to be highly expressed in human OSCC and its expression was suggested to act as a useful predictive and prognostic tool in clinical management of oral carcinomas. Herein the expression of HMGA2 and its sister gene HMGA1 were analysed within human and canine OSCC samples. Additionally, the HMGA negatively regulating miRNAs of the let-7 family as well as the let-7 regulating gene Lin28 were also comparatively analysed. Deregulations of either one of these members could affect the progression of human and canine OSCC.

**Methods:**

Expression levels of *HMGA1, HMGA2, Lin28, let-7a* and *mir-98* were analysed via relative qPCR in primary human and canine OSCC, thereof derived cell lines and non-neoplastic samples. Additionally, comparative HMGA2 protein expression was analysed by immunohistochemistry.

**Results:**

In both species, a significant up-regulation of the *HMGA2* gene was found within the neoplastic samples while *HMGA1* expression did not show significant deregulations. Comparative analyses showed down-regulation of mir-98 in human samples and up-regulation of let-7a and mir-98 in canine neoplastic samples. HMGA2 immunostainings showed higher intensities within the invasive front of the tumours than in the centre of the tumour in both species.

**Conclusions:**

HMGA2 could potentially serve as tumour marker in both species while HMGA1 might play a minor role in OSCC progression. Comparative studies indicate an inverse correlation of *HMGA2* and *mir-98* expression in human samples whereas in dogs no such characteristic could be found.

**Electronic supplementary material:**

The online version of this article (doi:10.1186/1471-2407-14-694) contains supplementary material, which is available to authorized users.

## Background

Oral cancer is the eighth most frequent cancer worldwide with even higher frequencies in developing than in developed countries [1]. Furthermore, men develop twice as frequent oral cancer than women and more than 95% of the carcinomas are of the squamous cell type. The standard treatment consists of surgery and/or radiation with additional chemotherapy in advanced stages of the disease. Tobacco and alcohol are regarded as the major risk factors for oral cancers but also infection with Human Papilloma Virus (HPV) are associated with a subset of head and neck cancers [2].

In dogs, oral cancer is the fourth most common cancer overall [3]. Similarly to humans, male dogs have a 2.4 times higher risk of developing oropharyngeal malignancies compared to female dogs and the tumours are staged similarly to those in humans but only 17-25% of the carcinomas are of the squamous cell type [3]. The risk for metastatic disease is site dependent with a higher metastatic potential in caudal tongue and tonsils and a lower metastatic rate in the rostral oral cavity [3]. Surgery and radiation are the most common treatment modalities and the mean survival time (MST) after surgery or radiation therapy is reported to lie between 26–36 months [3]. In humans, surgery and radiation are also the most common treatment modalities resulting in estimated overall 5-years survival rates for cancers of the oral cavity/pharynx and larynx between 58.3% and 64.5% [4–6].

Due to the heterogeneity of head and neck tumours with different site specific prognosis and survival, the integration of multiple selected prognostic tumour markers in association with the histopathologic features is advocated for risk assessment. The search for biomarkers includes evaluation of tumour tissues and surrogate material by molecular, genomic and phenotypic means [7].

The high mobility group (HMG) protein A family consists of two members *HMGA1* and *HMGA2* encoding thee major proteins: HMGA1a, HMGA1b, and HMGA2. The expression of *HMGA1* and *HMGA2* is high during embryogenesis and strongly reduced to very low, hardly detectable levels in adult tissues [8]. Re-expression in adult tissues was described in several human and canine neoplasias as cancers of the prostate and colorectum as well as lymphomas and non-small cell lung cancer [8–10].

Concerning human oral carcinoma, analysis of *HMGA2* expression was reported to be found significantly over-expressed in carcinoma tissues when compared to non neoplastic tissues [11]. Immunohistochemical localisation showed that HMGA2 protein was localised at the invasive front of oral carcinomas leading to the conclusion that HMGA2 immunostaining could be a prognostic determinant in stratifying patients into risk groups [11]. Analysis of HMGA1a and HMGA1b expression showed different findings reporting no significant expression deregulations [11] and increased expression in head and neck carcinomas, when compared to healthy mucosa samples [12].

*HMGA2* expression was shown to be partly regulated by the let-7 miRNA family member mir-98 in head and neck squamous cell carcinoma cell lines [13]. Studies analysing *HMGA* and let-7 expression in retinoblastomas and gastroenteropancreatic neuroendocrine tumors revealed a HMGA over expression accompanied by a down-regulation of let-7 [14, 15].

Let-7 micro RNAs themselves are regulated post-transcriptionally by the LIN28 and LIN28B proteins encoded by the *Lin28* gene [16–19]. Accordingly, over-expression of *Lin28* was found to be linked to a repression of let-7 family miRNAs and a combined down-regulation of let-7 and up-regulation of *Lin28* was reported in human neoplasias [20]. Interestingly a recent study analysing OSCCs reported increased expression of *Lin28a* and *Lin28b.*Thereby, the increased levels of Lin28b could be associated with poor prognosis [21].

In summary, the *Lin28* – let-7 –*HMGA* regulatory pathway and deregulations of either one of these members or of all involved proteins and miRNAs could have an effect on the progression and pathogeneses of human and canine OSCC. Thus, in our study we investigated the expression levels of *HMGA1, HMGA2, Lin28*, let-7 a and mir-98 via relative real time PCR in human and canine non-neoplastic and tumour tissue samples and human and canine cell lines which derived from primary OSCCs.

## Methods

### Tissue samples obtained from human patients

This study included human squamous cell carcinoma, non neoplastic controls, and tumour derived cell line samples which were obtained from 12 patients (9 male, 3 female, age 20–71 years) who underwent surgery at the Department of Oral and Maxillofacial Surgery, Hannover Medical School. Ethical approval and informed patient consent was obtained for all patients. This study was approved by the local ethics committee at the Hannover Medical School (Ref No. 984–2011). No patients had received preoperative chemotherapy or radiotherapy. The tumours (patients 2–12) were staged according to TNM staging system and were classified as follows: patient 2- pT4apN1, patient 3- pT1pN0, patient 4- rpT0 rpN2b R2 M1, patient 5- pT3pN0, patient 6- pT4apN0, patient 7- pT4apN1, patient 8- pT4apN2b, patient 9- pT4apN2c, patient 10- pT4apN2b, patient 11- pT2pN0, and patient 12-pT4 pN0.

### Tissue samples obtained from canine patients

Seven canine tumour and two healthy control samples (five female, four male) were used covering seven breeds: Boxer, Fox Terrier, Irish Terrier, Landseer, Retriever, Sheltie (n = 1 respectively), and three Mixed-breeds. Age ranged between a half year and eleven years. Samples derived from the maxilla (4), tongue (2), mandible (1), palate (1), and pharynx (1). All tumours were analysed immunohistologically. All diagnoses were cytologically and histologically confirmed according to the WHO Nomenclature. The tumours were staged and graded as follows: patient 3- grade IV (poor) stage T3bN1bM0, patient 4- grade I (well) stage T2aN0M0, patient 5- grade I (well) stage T3bN1aM0, patient 6- grade I (well) stage T3bN1bM0, patient 7- grade III (moderate) stage T3bN1bM0, patient 8- grade I (well) stage T1aN1bM0, patient 9- grade I (well) stage T2aN0M0. The non neoplastic control samples were collected from clinically unaltered tongue and palate tissues and the dogs were euthanized due to oral squamous cell carcinoma unrelated diseases. All samples were taken and provided by the Small Animal Clinic, University of Veterinary Medicine, Hannover, Germany according to the legislation of the state of Lower Saxony, Germany.

### Generation of canine and human cell lines

Due to the possibility to access fresh neoplastic material of both species we decided to aim at an establishment of OSCC cell lines as tools for further experimental approaches. The successful establishment of new cell lines allowed us to compare the gene expression patterns of the neoplastic primary tissues and the cell lines of both species. The respective human and canine tumour samples were verified to be squamous cell carcinomas by routine histopathologic characterisation. The samples were analysed by either a human or veterinary pathologist respectively. Two human cell lines were generated from freshly isolated squamous cell carcinoma biopsies derived from patient 4 and patient 12 (tumour staging see above). Single cell suspensions were prepared with a gentleMACS™ tissue dissociator (Miltenyi Biotec GmbH, Bergisch Gladbach, Germany). Samples were cut into small pieces of approximately 5 mm, transferred to a C Tube (Miltenyi Biotec GmbH, Bergisch Gladbach, Germany) containing 5 ml Dulbecco’s modified eagle medium (DMEM PAA, Pasching, Austria) and subjected to the first homogenisation step. After the addition of 1500 Units collagenase I (Cell Systems, St. Katharinen, Germany) and 0,5 mg neutral peptidase (Cell Systems, St. Katharinen, Germany), samples were incubated for 40 min at 37°C. Digested samples were subjected to a second homogenisation step followed by removal of tissue debris using a 70 μM cell strainer (BD Biosciences, Heidelberg, Germany). The cells were washed twice with culture medium (DMEM, 10% fetal calf serum, 20 mM Hepes, 1000 IU/ml penicillin and 0.1 mg/ml streptomycin; all PAA, Pasching, Austria) and plated on 100 mm cell culture dishes (Greiner, Frickenhausen, Germany) with DMEM and incubated at 37 C and 5% CO_2_ until confluent.

The canine cell line was generated from a freshly isolated oral squamous cell carcinoma biopsy. Due to the limited amount of bioptic material this sample was not used in the primary tissue screenings. The tumour tissue sample was cut into small pieces with a sterile scalpel and treated with collagenase (0.26%) for 2 hours at 37°C. The dissociated cells were transferred into a sterile 10 ml tube and centrifuged for 10 min at 1000*g. After centrifugation the supernatant was discarded and the resuspended cell pellet transferred into a sterile flask and incubated in 5 ml culture medium (Medium 199 (Invitrogen, Frankfurt, Germany), 10% fetal calf serum (PAA, Pasching, Austria) 200 U/ml penicillin and 200 ng/ml streptomycin (Biochrom, Berlin, Germany) and incubated at 37°C and 5% CO_2_ until confluent.

### Homogenisation of tissue samples and cell lysates of cultured cells

Tissue samples were homogenised using the stainless steel-beads and Qiagen-TissueLyser II homogeniser method accordingly to the manufacturer’s instructions (Qiagen, Hilden, Germany). Lysates of cultured cells were homogenised with QIAshredder columns accordingly to the manufacturer’s protocol (Qiagen, Hilden, Germany).

### RNA isolation and cDNA syntheses

RNA from tissue samples and cultured cells was isolated using the RNeasy Mini Kit according to the manufacturer’s instructions (Qiagen, Hilden, Germany). On-column DNase digestion was performed with the RNase-Free DNase set (Qiagen, Hilden, Germany). cDNA syntheses was performed using 250 ng RNA and the QuantiTect Reverse Transcription Kit following the manufacturer’s protocol (Qiagen, Hilden, Germany).

Furthermore, total RNA including small RNAs like miRNAs was isolated using the mirVana miRNA Isolation Kit according to the manufacturer’s instructions (Ambion, Applied Biosystems, Darmstadt, Germany). The respective cDNA syntheses were performed using 100 ng total RNA of each sample and the TaqMan MicroRNA Reverse Transcription Kit following the manufacturer’s protocol (Applied Biosystems, Darmstadt, Germany).

### *HMGA1*, *HMGA2, Lin28, GUSB*and *HPRT*real time PCR

Relative quantification real time PCRs for both species were carried out using the Eppendorf Mastercycler ep realplex real-time PCR System (Eppendorf AG, Hamburg, Germany).

For analysis of the human target genes, 2 μl of each cDNA was amplified in a total volume of 25 μl using universal PCR Mastermix and commercially purchased TaqMan gene Expression Assays (*HMGA1*– Assay ID: Hs00600784_g1; *HMGA2*– Assay ID: Hs00971724_m1; *Lin28A*- Assay ID: Hs04189307_g1; *HPRT*- Assay ID: Hs02800695_m1; *GUSB*- Assay ID: Hs99999808_m1; (Applied Biosystems, Darmstadt, Germany)).

For analysis of canine target genes, 2 μl of each cDNA was amplified using universal PCR Mastermix, self-designed TaqMan based Assays ([9, 22] (canine *HMGA1* (NM_001003387)- forward primer: 5′ ACCCAGTGAAGTGCCAACACCTAA 3′, reverse primer: 5′ CCTCCTTCTCCAGTTTTTTGGGTCT 3′, probe: 5′ 6-FAM-AGGGTGCTGCCAAGACCCGGAAAACTACCA-TAMRA 3′; canine *HMGA2* (DQ316099)- forward primer: 5′ AGTCCCTCCAAAGCAGCTCAAAAG 3′, reverse primer: 5′ GCCATTTCCTAGGTCTGCCTC 3′, probe: 5′ 6-FAM-CGCCCACTACTATGCCATCGTGTG-TAMRA 3′; canine *HPRT* (NM_001003357*)*- forward primer: 5′CCTTCTGCAGGAGAACCT 3′, reverse primer: 5′TCATCACTAATCACGACGCT 3′, probe: 5′6-FAM-CCTCCTGTTCAGGCTGCCGTCA-TAMRA 3′; canine *GUSB* (NM_001003191)- forward primer: 5′ TGGTGCTGAGGATTGGCA 3′, reverse primer: 5′ CTGCCACATGGACCCCATTC 3′, probe: 5′ 6-FAM-CGCCCACTACTATGCCATCGTGTG-TAMRA 3′) and commercially purchased TaqMan gene Expression Assays (Lin28- Assay ID: Cf02725509_g1 (Applied Biosystems, Darmstadt, Germany)).

The canine and human HMGA1 qPCR assays detected both splicing variants (HMGA1a and HMGA1b) simultaneously. PCR conditions were as follows: 10 min at 95°C, followed by 45 cycles with 15 s at 95°C and 1 min at 60°C. All human and canine samples were measured in triplicate and for each run non-template controls and non-reverse transcriptase control reactions were included.

### Let-7a, mir-98 and RNU6B real-time PCR

Relative quantification of the human and canine let-7a, mir-98 and RNU6B micro RNA transcript levels were carried out using 1.33 μl of each cDNA amplified in a total volume of 20 μl using TaqMan Universal PCR Master Mix, No AmpErase UNG and TaqMan MicroRNA assays for each gene (Let-7a- Assay ID: 000377; mir-98- Assay ID: 000577; RNU6B- Assay ID: 001093 (Applied Biosystems, Darmstadt, Germany)).

PCR conditions were as follows: 10 min at 95°C, followed by 45 cycles with 15 s at 95°C and 1 min at 60°C. All samples were measured in triplicate and for each run non-template controls and non-reverse transcriptase control reactions were included.

A precedent efficiency analysis of the microRNA PCR assays which were used in this study was performed by applying the same template and dilution steps.

### Histological and immunohistochemical procedures

The formalin-fixed specimens were embedded in paraffin, sectioned at 4 μm and routinely stained with haematoxylin and eosin (H&E). Thereafter, polyclonal goat-anti human HMGA2 (R&D Systems, Minneapolis, MN, USA) (1:400) or mouse-anti human Ki-67 antibody (Dianova, Hamburg, Germany) (1:100) was applied and allowed to incubate for approximately 16–18 h. Sections were incubated with biotin-conjugated horse antibody to goat IgG or goat anti-mouse IgG (both Vector Laboratories, Burlingame, CA, USA) (1:200) followed by ABC solution (Vectastain Elite ABC kit, Vector). Tyramine amplification reaction was performed according to the method of Adams (1992) [23] for 15 min (only HMGA2). The chromogen 3-amino-9-ethyl-carbazol (AEC) (Biologo, Kronshagen, Germany) was used for visualization followed by counterstaining with Mayer’s haematoxylin. Negative control sections were prepared by substituting the primary antibody with PBS. For the scoring, the percentage of carcinoma cells with intense red positive nuclear labelling for HGMA2 was estimated by examining the centre of the tumour and the invasive front (0: no expression; <25% weak expression; 25-50% moderate expression; >50%: strong expression).

### Statistical analysis

Statistical analysis of the relative real time PCR results applying the Hypothesis Test was performed with the Relative expression software tool REST 2008, version 2.0.7 [24]. A p-value of <0.05 was considered statistically significant.

## Results

### Real time PCR expression analyses of *HMGA1*and *HMGA2*

#### Human samples

**Figure 1:**
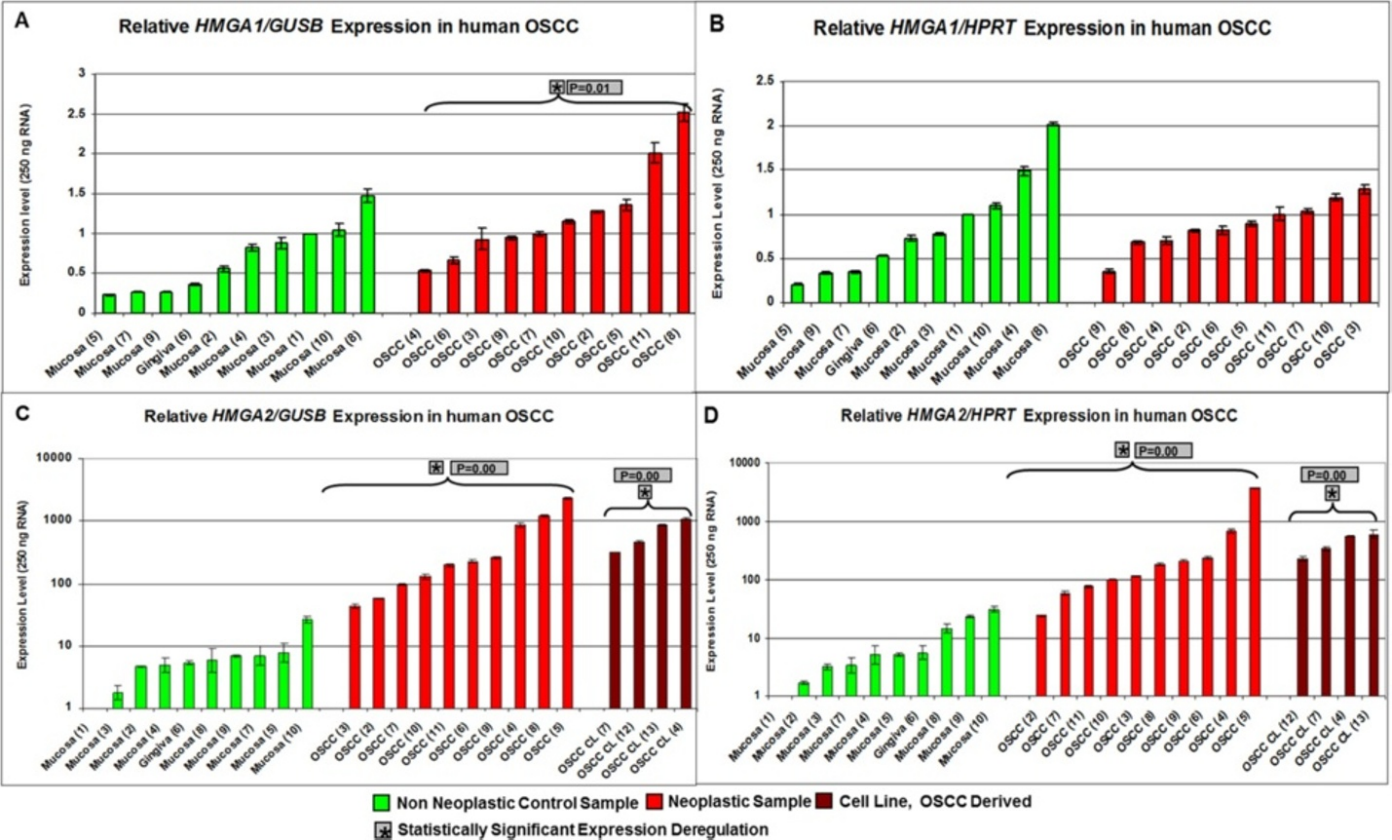
**Expression analyses of** ***HMGA1*** **and** ***HMGA2*** **in human OSCC.** The study included 10 non neoplastic control samples (green columns) and 10 tumour samples (red columns). **A**: relative *HMGA1/GUSB* real time PCR. **B**: relative *HMGA1/HPRT* real time PCR. **C**: relative *HMGA2/GUSB* real time PCR. **D**: relative *HMGA2/HPRT* real time PCR. Statistical analysis was performed applying the Hypothesis Test using REST 2008 (version 2.0.7.). * indicates a statistical significant expression deregulation of the *HMGA* genes when compared to non neoplastic control group; p-value is displayed next to *.

*HMGA1/GUSB* expression levels varied from 0.225 to 1.47 within the control samples, and from 0.53 to 2.52 within the tumour samples. The *HMGA1/HPRT* expression levels ranged from 0.21 to 2.02 within the control samples and from 0.36 to 1.28 within the tumour samples (details Figure 1A, B and Additional file 1: Table S1).

*HMGA2/GUSB* values ranged between 1 and 26.8 in the control samples, 44.4 and 2330 in the tumour samples, and 319 and 1092 within the cell culture samples. *HMGA2/HPRT* expression levels ranged between 1 and 31.1 within the control group, 24.1 and 3778 within the tumour group, and 226 to 561 within the cell culture samples (Figure 1C, D and Additional file 1: Table S1).

#### Canine samples

**Figure 2:**
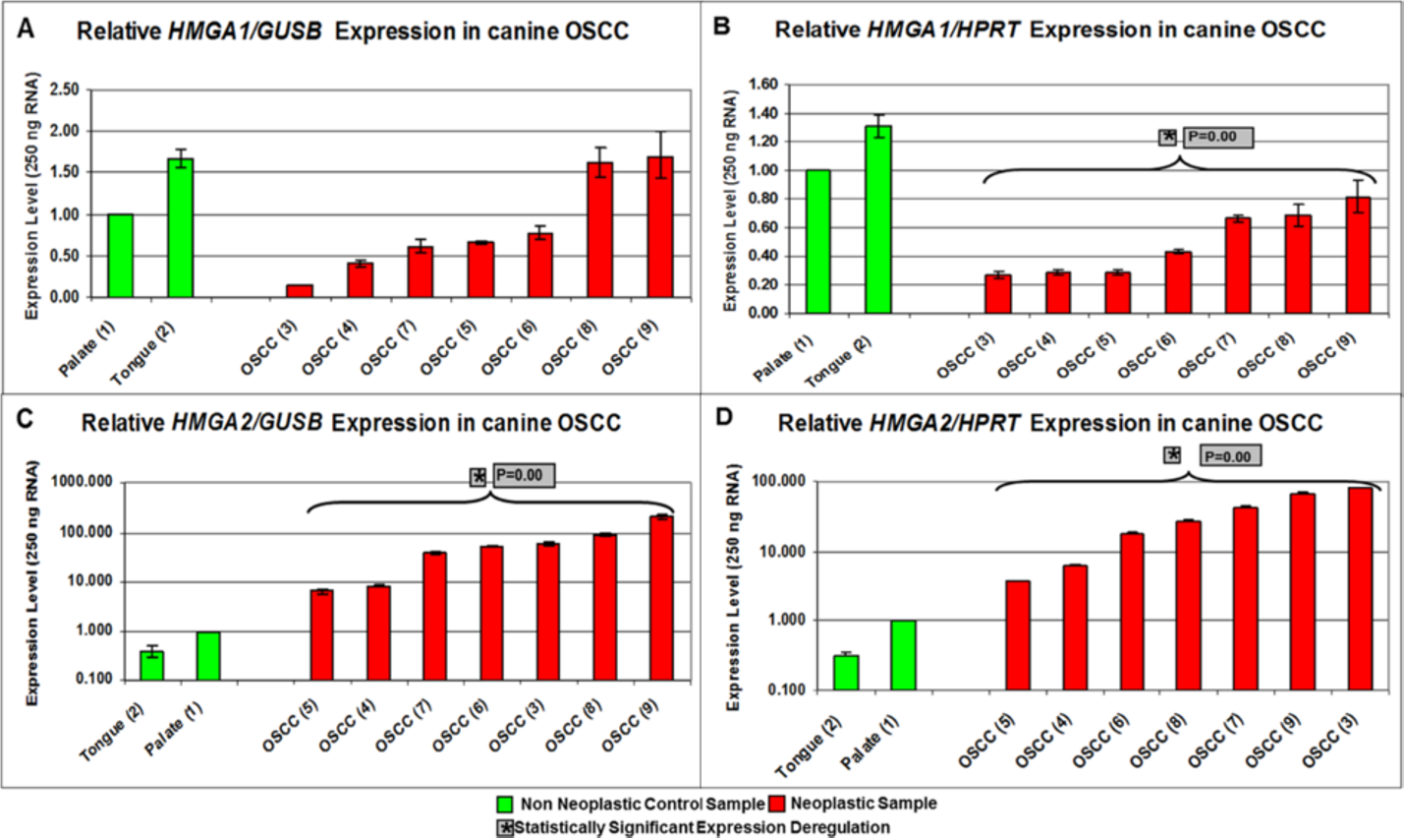
**Expression analyses of** ***HMGA1*** **and** ***HMGA2*** **in canine OSCC.** The study included 2 non neoplastic control samples (green columns) and 7 tumour samples (red columns). **A**: relative *HMGA1/GUSB* real time PCR. **B**: relative *HMGA1/HPRT* real time PCR. **C**: relative *HMGA2/GUSB* real time PCR. **D**: relative *HMGA2/HPRT* real time PCR. Statistical analysis was performed applying the Hypothesis Test using REST 2008 (version 2.0.7.). * indicates a statistical significant expression deregulation of the *HMGA* genes when compared to non neoplastic control group; p-value is displayed next to *.

*HMGA1/GUSB* expression levels varied from 1 to 1.67 within the non-neoplastic samples and from 0.152 to 1.69 within the neoplastic samples. *HMGA1/HPRT* expression ranged from 1 to 1.31 in the non neoplastic and 0.269 to 0.811 within the tumour samples (details Figure 2A, B and Additional file 2: Table S2).

*HMGA2/GUSB* expression levels ranged from 0.383 to 1 within the non neoplastic samples and from 6.45 to 208 within the neoplastic samples. *HMGA2/HPRT* expression ranged from 0.31 to 1 within the control group, and from 3.69 to 80.7 within the tumours (Figure 2C, D and Additional file 2: Table S2).

### Statistical analyses of *HMGA1*and *HMGA2*expression

#### Human samples

*HMGA1* was up-regulated when *GUSB* was used as endogenous control gene within the tumour samples (p = 0.0011) when compared to the non neoplastic samples. *HMGA2* was up-regulated when *GUSB* and *HPRT* were used as endogenous control genes within the tumour and cell line samples when compared to the non neoplastic samples.

The respective p-values of the tumour and cell line sample groups were p = 0.000 despite of the cell line sample group within the *HMGA2/GUSB* real time PCR showing a p-value of 0.001 (Figure 1).

#### Canine samples

*HMGA1* was down-regulated when *HPRT* was used as endogenous control gene within the tumour samples (p = 0.0011) compared to the non neoplastic control samples. *HMGA2* was up-regulated when *GUSB* and *HPRT* were used as endogenous control genes within the tumour samples when compared to the non neoplastic controls. The analysed p-values were p = 0.002 and p = 0.003 within the *HMGA2/GUSB* and *HMGA2/HPRT* real time PCRs respectively (Figure 2).

### Immunohistochemistry

#### Human tumour sections

**Figure 3:**
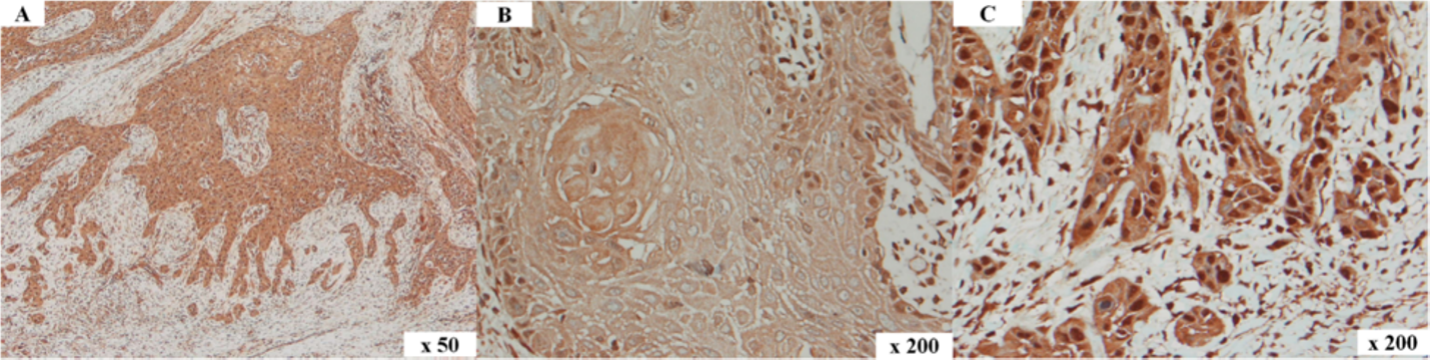
**HMGA2 immunohistochemistry in human OSCC.** Immunolabelling of a human tumour: overview **(A)**, tumour centre **(B)** and invasive front **(C)**. In the tumour centre **(B)** lower numbers of tumour cells with nuclear immunolabelling are present when compared to the respective invasive front **(C)**. The invasive front shows numerous tumour cells exhibiting intense nuclear immunolabelling of HMGA2. Magnification: **(A)** 50x, **(B)** and **(C)** 200x.

In the sections of patients 4 and 5 positive nuclear staining for HMGA2 was detected in the tumour cells that were located in the centre and/or the invasive front of the tumours (Figure 3A-C). The percentages of labelled tumour cells located in the centre were as follows: patient 4 – 30%, patient 5- 80%. Staining intensities of HMGA2 of tumour cells located at the invasive front were as follows: patient 4 – moderate (2+), patient 5 – strong (3+). The remaining analysed sections of patients analysed did not show positive staining for HMGA2.

#### Canine tumour sections

**Figure 4:**
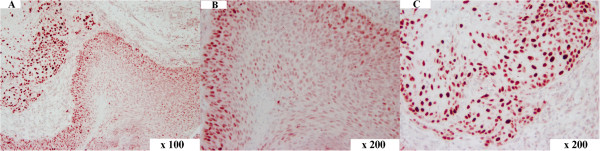
**HMGA2 immunohistochemistry in canine OSCC.** Immunolabelling of a canine tumour grade II: overview **(A)**, tumour centre **(B)** and invasive front **(C)**. HMGA2 staining in the tumour centre **(B)** revealed approx. 25% tumour cells with nuclear immunolabelling while cells at the invasive front showed approx. 50% staining **(C)**. Magnification: **(A)** 100x, **(B)** and **(C)** 200x.

In all sections positive nuclear staining for HMGA2 was detected in the tumour cells that were located in the centre and/or at the invasive front of the tumours as well (Figure 4). The percentages of labelled tumour cells located in the centre were as follows: patients 3, 4, 7, 8 – 25-50% respectively. Patients 5, 6 and 9 - <25% respectively. The percentages of labelled tumour cells located at the invasive front were as follows: patient 3, 5, 6, 8 – 25-50%, patient 4, 7 and 9 - >50%, respectively. Except the cases of patients 3 and 8, higher percentages of positive nuclei were found at the invasive front of the tumours. The percentages of Ki-67 labelled tumour cells at the invasive front varied between 36% and 68%. In the decalcified sample from patient 6, no Ki-67 labelling was seen.

### Comparative expression analyses of *HMGA2*, *Lin28*, let-7a and mir-98

#### Human samples

**Figure 5:**
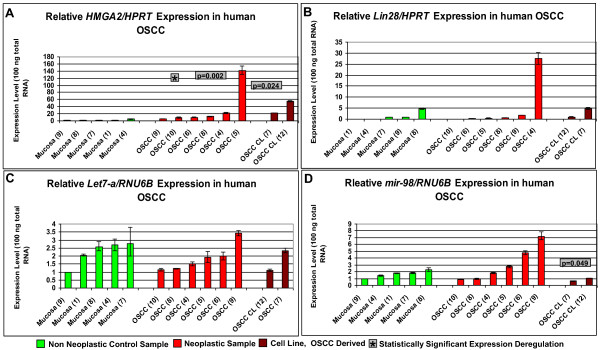
**Comparative expression analyses of the** ***HMGA2*** **and** ***Lin28*** **genes and the let-7a and mir-98 miRNAs in human OSCC.** The study included 5 non neoplastic control samples (green columns), 6 tumour samples (red columns) and 2 patient derived cell lines (brown columns). **A**: relative *HMGA2/HPRT* real time PCR. **B**: relative *Lin28/HPRT* real time PCR. **C**: relative *let-7a/RNU6B* real time PCR. **D**: relative *mir-98/RNU6B* real time PCR. Statistical analysis of the relative real time PCR results (Hypothesis Test) was performed with REST 2008 software tool. A p-value of <0.05 was considered statistically significant. * indicates a statistical significant expression deregulation of *HMGA2* and/or *Lin28* and/or *let-7a* and/or *mir-98* when compared to non neoplastic control group; p-value is displayed next to *.

Relative *HMGA2/HPRT* expression levels ranged from 1 to 4.61 within the non neoplastic samples, from 5.92 to 141 within the neoplastic samples and from 22.2 to 55.1 within the cell line samples (Figure 5A, Additional file 3: Table S3).

*Lin28/HPRT* expression varied between 0 and 4.65 within the non neoplastic samples, from 0 to 27.5 within the neoplastic samples and from 1.03 to 4.87 (patient 7) within the cell lines (Figure 5B, Additional file 3: Table S3).

let-7a/RNU6B expression ranged from 1 to 2.76 within the non neoplastic samples, from 1.14 to 3.42 within the neoplastic samples and from 1.11 to 2.33 within the cell lines (Figure 5C, Additional file 3: Table S3).

mir-98/RNU6B expression ranged from 1 to 2.28 within the non neoplastic samples, from 0.91 to 7.22 within the neoplastic and from 0.65 to 1.05 within the cell lines (Figure 5D, Additional file 3: Table S3).

#### Canine samples

**Figure 6:**
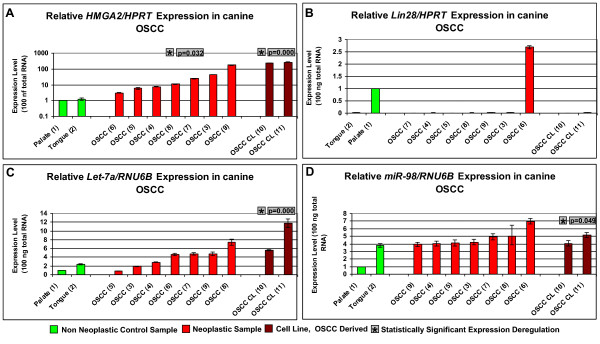
**Comparative expression analyses of the** ***HMGA2*** **and** ***Lin28*** **genes and the let-7a and mir-98 miRNAs in canine OSCC.** The study included 2 non neoplastic control samples (green columns), 7 tumour samples (red columns) and 2 cell line derived samples (brown columns) which derived from patients 1–10. **A**: relative *HMGA2/HPRT* real time PCR. **B**: relative *Lin28/HPRT* real time PCR. **C**: relative *let-7a/RNU6B* real time PCR. **D**: relative *mir-98/RNU6B* real time PCR. Statistical analysis of the relative real time PCR results (Hypothesis Test) was performed with REST 2008 software tool. A p-value of <0.05 was considered statistically significant. * indicates a statistical significant expression deregulation of *HMGA2* and/or *Lin28* and/or *let-7a* and/or *mir-98* when compared to non neoplastic control group; p-value is displayed next to *.

*HMGA2/HPRT* expression ranged from 1 to 1.19 within the non neoplastic samples, from 3.09 to 178 within the neoplastic samples and from 242 to 270 within the cell line (Figure 6A, Additional file 4: Table S4).

*Lin28/HPRT* expression ranged between 0.02 and 1 within the non neoplastic samples, between 0 and 6.97 within the neoplastic samples, and between 0 and 0.03 within the cell line (Figure 6B, Additional file 4: Table S4).

let7-a/RNU6B expression levels ranged from 1 to 2.32 within the non neoplastic samples, from 0.84 to 7.36 within the neoplastic samples and from 5.6 to 11.7 within the cell line samples (Figure 6C, Additional file 4: Table S4).

Mir-98/RNU6B expression ranged from 1 to 3.8 within the non neoplastic samples, from 3.93 to 6.97 within the neoplastic samples and from 4.06 to 5.18 within the cell line (Figure 6D, Additional file 4: Table S4).

### Statistical analyses of comparative *HMGA2, Lin28,*let-7a and mir-98 expression

#### Human samples

*HMGA2* was up-regulated when *HPRT* was used as endogenous control gene within the tumour (p = 0.002) and cell line samples (p = 0.024) when compared to the non neoplastic samples (Figure 5A). mir-98 was down-regulated when RNU6B was used as endogenous control within the cell line samples (p = 0.049) when compared to the non neoplastic control samples (Figure 5D).

#### Canine samples

*HMGA2* was up-regulated when *HPRT* was used as endogenous control gene within the tumour (p = 0.032) and cell line samples (p = 0.000) when compared to the non neoplastic samples (Figure 6A). let7-a was up-regulated when RNU6B was used as endogenous control within the cell lines samples (p = 0.000) when compared to the non neoplastic control samples (Figure 6C). mir-98 was up-regulated when RNU6B was used as endogenous control within the tumour samples (p = 0.000) when compared to the non neoplastic control samples (Figure 6D).

## Discussion

The evaluation of the *HMGA* gene expression in the analysed human and canine neoplasias showed consistent expression in both species. Whereas *HMGA2* was found to be strongly upregulated in the primary samples as well as in the analysed cell lines the sister gene *HMGA1* showed expression variation ranging from two to three fold. Thus, herein *HMGA2* expression shows potential marker characteristics in both species whereas *HMGA1* misses the required characteristics.

Previous studies analysing the *HMGA1* expression in human head and neck cancers reported contradictory results, showing no statistical significant deregulations as well as increased expression of *HMGA1* in OSCC [11, 12], matching our results in both species. The herein characterised high *HMGA2* expression in the neoplastic samples of both species, affirms the findings by Miyazawa et al. [11] reporting *HMGA2* over-expression of 84-315-fold in analysed carcinoma tissues when compared to non neoplastic tissues [11].

HMGA2 was found to be expressed at the invasive front of oral carcinomas leading to the conclusion that –in contrast to HMGA1- HMGA2 immunostaining could be a potential prognostic determinant in stratifying patients into risk groups [11]. Further, multivariate risk factor analysis demonstrated that HMGA2 expression was found to be a significant independent predictor of death of carcinoma and as an independent prognostic marker for disease-specific overall survival [11]. Contrary to this, HMGA1 expression was also reported to be increased in head and neck carcinomas analysed via semi-quantitative RT-PCR and immunohistochemistry when compared to healthy mucosa samples [12].

The herein reported immunohistochemical staining in human sections showed positive HMGA2 staining within the centre and the invasive front of the tumour sections in only two patients. In contrast, in all canine samples the immunohistochemichal staining showed positive signals for HMGA2. Thereby, with exception of two cases, the percentages of positive nuclei for HMGA2 were higher at the invasive front of the tumour sections than in the centre of the tumours. Similarly, Miyazawa et al. reported a negligible HMGA2 staining in the central area of the human carcinoma tissues whereas it was ectopically expressed at the invasive front [11]. In general, most of the oral cancer deaths result from local invasion and distant metastasis. At the invasive front, the carcinoma cells gain the characteristics similar to mesenchymal cells due to epithelial-mesenchymal transition (EMT) where cells switch from a polarised epithelial phenotype to a motile mesenchymal phenotype and facilitate tumour invasion [25, 26]. The role of HMGA2, which is a mesenchym-specific factor was thus strongly correlated with poor differentiation, invasion and metastasis of oral cancer [11, 25, 26]. Our immunohistochemical findings in canine OSCC are comparable with the findings seen in human OSCC supporting the data discussing a possible role of HMGA2 as a factor promoting cell proliferation and motility at the invasive front in canine oral cancer.

In our study, mir-98 was significantly down-regulated in the analysed OSCC cell lines describing an inverse expression pattern of *HMGA2* and mir-98. As this observation is limited to qPCR assays, a definitive statement can hardly be drawn at this point without further transcriptomic analyses. However, interestingly Hammond et al. postulated a linear pathway from Lin28 to let-7 to HMGA2 [27]. In embryonic stem cells Lin28 expression is highly leading to the inhibition of let-7 microRNAs processing steps. These low let-7 microRNAs levels allow a high expression of their usually repressed target genes as HMGA2. Accordingly, HMGA2 expression increases as observed in embryonic stem cells and in several cancers [27]. Our results are in agreement with a study by Hebert et al. reporting that *HMGA2* expression is partly regulated by mir-98 in head and neck squamous cell carcinoma cell lines [13]. The let-7a miRNA was reported to be down-regulated in head and neck cancer tissues and the expression levels were significantly reduced in metastatic tissues when compared to primary tumours. Thereby, a high let-7a score was found to be associated with early T-stage and low lymph node metastasis and early pathological stage [28]. Additionally, in human oesophageal squamous cell carcinoma, an inverse transcription of let-7 and HMGA2 was reported [29]. However, the authors did not mention which members of the let-7 family were analysed and thus it cannot be excluded that let-7a and mir-98 were not analysed within the study.

We performed the statistical analysis of the different real time PCRs for all samples as groups with no regard to the single patients. The human patients 4 and 7 could provide real time data for both sample types as non-neoplastic and neoplastic material was existent. Expression of *HMGA2* and *Lin28* were higher in the neoplastic samples while let-7a and mir-98 expression were higher in the non-neoplastic samples. Here, an inverse correlation of the expression of *HMGA2* and *Lin28* and let-7a and mir-98 could be detected in the tumour samples when compared to healthy tissue. These results strongly fortify the described *Lin28* to let-7 to *HMGA2 axis* hypothesis drawn by Hammond et al. [27]. Further, increased levels of *Lin28b* could be associated with poor prognosis OSCCs [21]. Nevertheless, it must be considered that the statistical analyses of our real time PCRs did not confirm a significant down-regulation of let-7a and a simultaneous over-expression of *Lin28* when all samples were analysed as groups. In general, an inverse correlation of let-7 and *HMGA2* is a frequent finding in many types of human neoplasias. Nevertheless, in adipocytic tumours, despite of some individual let-7 miRNAs, no global correlation between expression of let-7 members and HMGA2 were detected [30]. Similarly, our results regarding let-7a expression suggest, that a decrease in let-7a expression does not appear to be main mechanism accounting for *HMGA2* deregulation. Hereby, let-7a is likely to be involved in the pathogenesis of OSCC probably through mechanisms other than those expected.

Nevertheless, our results provide a first trend of comparative expression of *HMGA2, Lin28,* let-7a and mir-98 in OSCC and must be verified in a larger set of tissue control and tumour samples to determine if there is an inverse correlation of expression between these genes and if there is a potential link to disease progression.

Comparative expression analyses of *HMGA2, Lin28*, let-7a and mir-98 in canine samples showed a statistical significant over expression of *HMGA2* in neoplastic samples confirming our precedent results. Besides this, the study revealed different results when compared to the results made in the comparative expression study in human samples. Let-7a was over expressed within the cell line samples while mir-98 was over expressed in the tumour samples. These findings do not indicate a negative correlation of expression of *HMGA2* and *Lin28* and the let-7a and mir-98. Thus, analysis of more control and tumour samples would be required to improve the power of the study and additional microRNAs should be taken into account, which might be involved in the progression of canine OSCC.

A correlation between the analysed targets and the survival time was not focussed in this study due to the limited number of analysed cases. Additionally, regarding canine patients, an accurate follow up is often hindered by the fact that veterinary patients are frequently not represented at the academic institution as owners follow treatment at local veterinarians. Furthermore, central cancer register for canine patients, as present for human cancer patients, does not exist. However, due to the similarities in canine and human cancer presentation, as reported herein, basic research and the development of clinical regimens in either of the species provide valuable solid data for the respective counterpart.

## Conclusion

In conclusion, the comparative expression study analysing a possible inverse correlation between *HMGA2* and *Lin28* and let-7 members in OSCC revealed a down-regulation of mir-98 with a simultaneous *HMGA2* over expression in human OSCC cell line samples. In contrast to the findings made in human samples, comparative analyses in canine showed different expression patterns. Despite the *HMGA2* over-expression in neoplastic samples, the miRNAs let-7a and mir-98 were found to be up-regulated. Consequently, in canine OSCC further factors might be involved in the progression neoplasia when compared to the analysed human OSCCs. Furthermore the miRNAs analysed herein might not reflect the main factors involved in deregulation of *HMGA2* in canine OSCC.

## Electronic supplementary material

Additional file 1: Table S1: Expression analyses of *HMGA1* and *HMGA2* in human OSCC. Relative real-time PCR reactions were performed with human *GUSB* and *HPRT* as endogenous control genes. The non neoplastic mucosa sample obtained from patient 1 was used for calibration during data analyses. (DOC 130 KB)

Additional file 2: Table S2: Expression analyses of *HMGA1* and *HMGA2* in canine OSCC. Relative real-time PCR reactions were performed with canine *GUSB* and *HPRT* as endogenous control genes. The non neoplastic palate sample obtained from patient 1 was used for calibration during data analyses. (DOC 62 KB)

Additional file 3: Table S3: Comparative expression analyses of the *HMGA2* and *Lin28* genes and the let-7a and mir-98 miRNAs in human OSCC Relative real-time PCR reactions were performed with human *HPRT* and RNU6B as endogenous control genes. The non neoplastic mucosa sample obtained from patient 9 was used for calibration during data analyses. (DOC 74 KB)

Additional file 4: Table S4: Comparative expression analyses of the *HMGA2* and *Lin28* genes and the let-7a and mir-98 miRNAs in canine OSCC Relative real-time PCR reactions were performed with canine *HPRT* and RNU6B as endogenous control genes. The non neoplastic palate sample obtained from patient 1 was used for calibration during data analyses. (DOC 60 KB)
