# Supplementary material for: HMGA1 and HMGA2 expression and comparative analyses of HMGA2, Lin28 and let-7 miRNAs in oral squamous cell carcinoma
Source: BMC Cancer. 2014 Sep 23;14:694. doi: 10.1186/1471-2407-14-694 (PMC4190370; doi:10.1186/1471-2407-14-694)
Supplement: Supplementary file 1 — Additional file 1: Table S1: Expression analyses of HMGA1 and HMGA2 in human OSCC. Relative real-time PCR reactions were performed with human GUSB and HPRT as endogenous control genes. The non neoplastic mucosa sample obtained from patient 1 was used for calibration during data analyses. (DOC 130 KB) [file 12885_2013_4893_MOESM1_ESM.doc]

| **Patient**  Sample | **Real-time PCR:**  Target gene / Endogenous control gene | **Expression**  **level** | **SD+** | **SD-** |
| --- | --- | --- | --- | --- |
| **1**  Healthy mucosa | *HMGA1 / GUSB* | 1 | 0 | 0 |
|  | *HMGA1 / HPRT* | 1 | 0 | 0 |
|  | *HMGA2 / GUSB* | 1 | 0 | 0 |
|  | *HMGA2 / HPRT* | 1 | 0 | 0 |
| **2**  Healthy mucosa | *HMGA1 / GUSB* | 0.56 | 0.04 | 0.04 |
|  | *HMGA1 / HPRT* | 0.73 | 0.03 | 0.03 |
|  | *HMGA2 / GUSB* | 4.77 | 0.08 | 0.08 |
|  | *HMGA2 / HPRT* | 1.73 | 0.1 | 0.09 |
| **2**  Tumour | *HMGA1 / GUSB* | 1.28 | 0.01 | 0.02 |
|  | *HMGA1 / HPRT* | 0.81 | 0.02 | 0.02 |
|  | *HMGA2 / GUSB* | 58.6 | 1.8 | 1.8 |
|  | *HMGA2 / HPRT* | 24.1 | 1 | 0.9 |
| **3**  Healthy mucosa | *HMGA1 / GUSB* | 0.88 | 0.07 | 0.07 |
|  | *HMGA1 / HPRT* | 0.77 | 0.02 | 0.02 |
|  | *HMGA2 / GUSB* | 1.78 | 0.53 | 0.4 |
|  | *HMGA2 / HPRT* | 3.24 | 0.37 | 0.34 |
| **3**  Tumour | *HMGA1 / GUSB* | 0.93 | 0.14 | 0.13 |
|  | *HMGA1 / HPRT* | 1.28 | 0.05 | 0.05 |
|  | *HMGA2 / GUSB* | 44.4 | 3.2 | 2.9 |
|  | *HMGA2 / HPRT* | 117 | 3 | 4 |
| **4**  Healthy mucosa | *HMGA1 / GUSB* | 0.82 | 0.04 | 0.04 |
|  | *HMGA1 / HPRT* | 1.49 | 0.04 | 0.05 |
|  | *HMGA2 / GUSB* | 5.01 | 1.53 | 1.17 |
|  | *HMGA2 / HPRT* | 5.18 | 2.42 | 1.65 |
| **4**  Tumour | *HMGA1 / GUSB* | 0.53 | 0.02 | 0.02 |
|  | *HMGA1 / HPRT* | 0.7 | 0.04 | 0.04 |
|  | *HMGA2 / GUSB* | 867 | 52 | 50 |
|  | *HMGA2 / HPRT* | 682 | 63 | 57 |
| **4**  Tumour derived Cell line | *HMGA2 / GUSB* | 1092 | 38 | 37 |
|  | *HMGA2 / HPRT* | 561 | 5 | 4 |
| **5**  Healthy mucosa | *HMGA1 / GUSB* | 0.23 | 0.01 | 0.01 |
|  | *HMGA1 / HPRT* | 0.21 | 0.01 | 0.01 |
|  | *HMGA2 / GUSB* | 7.78 | 3.55 | 2.25 |
|  | *HMGA2 / HPRT* | 5.23 | 0.35 | 0.33 |
| **5**  Tumour | *HMGA1 / GUSB* | 1.36 | 0.07 | 0.07 |
|  | *HMGA1 / HPRT* | 0.89 | 0.03 | 0.03 |
|  | *HMGA2 / GUSB* | 2330 | 115 | 109 |
|  | *HMGA2 / HPRT* | 3778 | 109 | 106 |
| **6**  Healthy gingiva | *HMGA1 / GUSB* | 0.36 | 0.01 | 0.01 |
|  | *HMGA1 / HPRT* | 0.53 | 0.01 | 0.01 |
|  | *HMGA2 / GUSB* | 5.41 | 0.35 | 0.33 |
|  | *HMGA2 / HPRT* | 5.58 | 1.85 | 1.39 |
| **6**  Tumour | *HMGA1 / GUSB* | 0.67 | 0.04 | 0.04 |
|  | *HMGA1 / HPRT* | 0.81 | 0.04 | 0.04 |
|  | *HMGA2 / GUSB* | 231 | 17 | 15 |
|  | *HMGA2 / HPRT* | 243 | 12 | 10 |
| **7**  Healthy mucosa | *HMGA1 / GUSB* | 0.26 | 0.01 | 0.01 |
|  | *HMGA1 / HPRT* | 0.35 | 0.02 | 0.02 |
|  | *HMGA2 / GUSB* | 7.09 | 2.87 | 2.05 |
|  | *HMGA2 / HPRT* | 3.44 | 1.28 | 0.94 |
| **7**  Tumour | *HMGA1 / GUSB* | 0.99 | 0.03 | 0.03 |
|  | *HMGA1 / HPRT* | 1.04 | 0.03 | 0.03 |
|  | *HMGA2 / GUSB* | 98.6 | 4.4 | 3.8 |
|  | *HMGA2 / HPRT* | 59.1 | 4.7 | 4.3 |
| **7**  Tumour derived Cell line | *HMGA2 / GUSB* | 319 | 6 | 4 |
|  | *HMGA2 / HPRT* | 341 | 27 | 25 |
| **8**  Healthy mucosa | *HMGA1 / GUSB* | 1.47 | 0.09 | 0.09 |
|  | *HMGA1 / HPRT* | 2.02 | 0.03 | 0.02 |
|  | *HMGA2 / GUSB* | 5.92 | 3.26 | 2.11 |
|  | *HMGA2 / HPRT* | 14.6 | 2.7 | 2.4 |
| **8**  Tumour | *HMGA1 / GUSB* | 2.52 | 0.11 | 0.11 |
|  | *HMGA1 / HPRT* | 0.68 | 0.02 | 0.02 |
|  | *HMGA2 / GUSB* | 1219 | 62 | 59 |
|  | *HMGA2 / HPRT* | 185 | 5 | 5 |
| **9**  Healthy mucosa | *HMGA1 / GUSB* | 0.26 | 0.01 | 0.01 |
|  | *HMGA1 / HPRT* | 0.33 | 0.01 | 0.01 |
|  | *HMGA2 / GUSB* | 7.02 | 0.31 | 0.3 |
|  | *HMGA2 / HPRT* | 23.7 | 1.2 | 1.2 |
| **9**  Tumour | *HMGA1 / GUSB* | 0.94 | 0.02 | 0.02 |
|  | *HMGA1 / HPRT* | 0.36 | 0.02 | 0.02 |
|  | *HMGA2 / GUSB* | 267 | 10 | 10 |
|  | *HMGA2 / HPRT* | 215 | 6 | 5 |
| **10**  Healthy mucosa | *HMGA1 / GUSB* | 1.04 | 0.09 | 0.07 |
|  | *HMGA1 / HPRT* | 1.09 | 0.03 | 0.03 |
|  | *HMGA2 / GUSB* | 26.8 | 2.9 | 2.7 |
|  | *HMGA2 / HPRT* | 31.1 | 4.1 | 3.6 |
| **10**  Tumour | *HMGA1 / GUSB* | 1.15 | 0.02 | 0.02 |
|  | *HMGA1 / HPRT* | 1.19 | 0.04 | 0.04 |
|  | *HMGA2 / GUSB* | 131 | 11 | 10 |
|  | *HMGA2 / HPRT* | 102 | 2 | 2 |
| **11**  Tumour | *HMGA1 / GUSB* | 2.01 | 0.12 | 0.12 |
|  | *HMGA1 / HPRT* | 1 | 0.08 | 0.07 |
|  | *HMGA2 / GUSB* | 199 | 9 | 9 |
|  | *HMGA2 / HPRT* | 76.9 | 2 | 1.9 |
| **12**  Tumour derived Cell line | *HMGA2 / GUSB* | 467 | 19 | 19 |
|  | *HMGA2 / HPRT* | 226 | 20 | 18 |
| **13**  Tumour derived Cell line | *HMGA2 / GUSB* | 863 | 35 | 34 |
|  | *HMGA2 / HPRT* | 611 | 106 | 90 |
