# Supplementary material for: HMGA1 and HMGA2 expression and comparative analyses of HMGA2, Lin28 and let-7 miRNAs in oral squamous cell carcinoma
Source: BMC Cancer. 2014 Sep 23;14:694. doi: 10.1186/1471-2407-14-694 (PMC4190370; doi:10.1186/1471-2407-14-694)
Supplement: Supplementary file 2 — Additional file 2: Table S2: Expression analyses of HMGA1 and HMGA2 in canine OSCC. Relative real-time PCR reactions were performed with canine GUSB and HPRT as endogenous control genes. The non neoplastic palate sample obtained from patient 1 was used for calibration during data analyses. (DOC 62 KB) [file 12885_2013_4893_MOESM2_ESM.doc]

| **Canine Patient**  Sample | **Real-time PCR:**  Target gene / Endogenous control gene | **Expression**  **level** | **SD+** | **SD-** |
| --- | --- | --- | --- | --- |
| **1**  Healthy palate | *HMGA1 / GUSB* | 1 | 0 | 0 |
|  | *HMGA1 / HPRT* | 1 | 0 | 0 |
|  | *HMGA2 / GUSB* | 1 | 0 | 0 |
|  | *HMGA2 / HPRT* | 1 | 0 | 0 |
| **2**  Healthy tongue | *HMGA1 / GUSB* | 1.67 | 0.12 | 0.11 |
|  | *HMGA1 / HPRT* | 1.31 | 0.08 | 0.08 |
|  | *HMGA2 / GUSB* | 0.383 | 0.12 | 0.09 |
|  | *HMGA2 / HPRT* | 0.315 | 0.04 | 0.03 |
| **3**  Tumour | *HMGA1 / GUSB* | 0.152 | 0.00 | 0.00 |
|  | *HMGA1 / HPRT* | 0.269 | 0.02 | 0.02 |
|  | *HMGA2 / GUSB* | 57.4 | 4.00 | 3.90 |
|  | *HMGA2 / HPRT* | 80.7 | 2.80 | 2.70 |
| **4**  Tumour | *HMGA1 / GUSB* | 0.40 | 0.04 | 0.04 |
|  | *HMGA1 / HPRT* | 0.28 | 0.02 | 0.02 |
|  | *HMGA2 / GUSB* | 8.29 | 0.27 | 0.26 |
|  | *HMGA2 / HPRT* | 6.22 | 0.25 | 0.24 |
| **5**  Tumour | *HMGA1 / GUSB* | 0.66 | 0.02 | 0.02 |
|  | *HMGA1 / HPRT* | 0.28 | 0.02 | 0.02 |
|  | *HMGA2 / GUSB* | 6.45 | 0.66 | 0.61 |
|  | *HMGA2 / HPRT* | 3.69 | 0.18 | 0.17 |
| **6**  Tumour | *HMGA1 / GUSB* | 0.78 | 0.08 | 0.08 |
|  | *HMGA1 / HPRT* | 0.43 | 0.02 | 0.02 |
|  | *HMGA2 / GUSB* | 52 | 2.20 | 2.20 |
|  | *HMGA2 / HPRT* | 18 | 0.70 | 0.60 |
| **7**  Tumour | *HMGA1 / GUSB* | 0.15 | 0.00 | 0.00 |
|  | *HMGA1 / HPRT* | 0.66 | 0.02 | 0.03 |
|  | *HMGA2 / GUSB* | 38 | 2.40 | 2.20 |
|  | *HMGA2 / HPRT* | 43.3 | 0.50 | 0.40 |
| **8**  Tumour | *HMGA1 / GUSB* | 1.62 | 0.19 | 0.17 |
|  | *HMGA1 / HPRT* | 0.68 | 0.08 | 0.07 |
|  | *HMGA2 / GUSB* | 90.6 | 4.60 | 4.40 |
|  | *HMGA2 / HPRT* | 27.3 | 0.60 | 0.60 |
| **9**  Tumour | *HMGA1 / GUSB* | 1.69 | 0.31 | 0.26 |
|  | *HMGA1 / HPRT* | 0.81 | 0.12 | 0.10 |
|  | *HMGA2 / GUSB* | 208 | 23.0 | 20.0 |
|  | *HMGA2 / HPRT* | 67.1 | 1.80 | 1.80 |
