# Supplementary material for: HMGA1 and HMGA2 expression and comparative analyses of HMGA2, Lin28 and let-7 miRNAs in oral squamous cell carcinoma
Source: BMC Cancer. 2014 Sep 23;14:694. doi: 10.1186/1471-2407-14-694 (PMC4190370; doi:10.1186/1471-2407-14-694)
Supplement: Supplementary file 3 — Additional file 3: Table S3: Comparative expression analyses of the HMGA2 and Lin28 genes and the let-7a and mir-98 miRNAs in human OSCC Relative real-time PCR reactions were performed with human HPRT and RNU6B as endogenous control genes. The non neoplastic mucosa sample obtained from patient 9 was used for calibration during data analyses. (DOC 74 KB) [file 12885_2013_4893_MOESM3_ESM.doc]

| **Patient**  Sample | **Real-time PCR:**  Target gene / Endogenous control gene | **Expression**  **level** | **SD+** | **SD-** |
| --- | --- | --- | --- | --- |
| **1**  Healthy mucosa | *HMGA2 / HPRT* | 1.75 | 0.49 | 0.38 |
|  | *Lin28 / HPRT* | 0 | 0 | 0 |
|  | *Let-7a / RNU6B* | 2.06 | 0.07 | 0.08 |
|  | *mir-98 / RNU6B* | 1.81 | 0.05 | 0.04 |
| **4**  Healthy mucosa | *HMGA2 / HPRT* | 4.61 | 0.26 | 0.25 |
|  | *Lin28 / HPRT* | 0 | 0 | 0 |
|  | *Let-7a / RNU6B* | 2.68 | 0.38 | 0.34 |
|  | *mir-98 / RNU6B* | 1.41 | 0.11 | 0.1 |
| **4**  Tumour | *HMGA2 / HPRT* | 21.2 | 2 | 1.8 |
|  | *Lin28 / HPRT* | 27.5 | 2.9 | 2.5 |
|  | *Let-7a / RNU6B* | 1.5 | 0.14 | 0.12 |
|  | *miRr98 / RNU6B* | 1.79 | 0.11 | 0.11 |
| **5**  Tumour | *HMGA2 / HPRT* | 141 | 13 | 11 |
|  | *Lin28 / HPRT* | 0.305 | 0.2 | 0.12 |
|  | *Let-7a / RNU6B* | 1.92 | 0.38 | 0.31 |
|  | *mir-98 / RNU6B* | 2.76 | 0.13 | 0.13 |
| **6**  Tumour | *HMGA2 / HPRT* | 9.8 | 0.9 | 0.82 |
|  | *Lin28 / HPRT* | 0.184 | 0.249 | 0.11 |
|  | *Let-7a / RNU6B* | 1.99 | 0.25 | 0.23 |
|  | *mir-98 / RNU6B* | 4.78 | 0.32 | 0.3 |
| **7**  Healthy mucosa | *HMGA2 / HPRT* | 1.59 | 0.23 | 0.19 |
|  | *Lin28 / HPRT* | 0.81 | 0.04 | 0.04 |
|  | *Let-7a / RNU6B* | 2.76 | 1.02 | 0.74 |
|  | *mir-98 / RNU6B* | 1.82 | 0.08 | 0.07 |
| **7**  Tumour derived Cell line | *HMGA2 / HPRT* | 22.2 | 0.7 | 0.7 |
|  | *Lin28 / HPRT* | 4.87 | 0.58 | 0.52 |
|  | *Let-7a / RNU6B* | 2.33 | 0.14 | 0.13 |
|  | *mir-98 / RNU6B* | 0.65 | 0.04 | 0.03 |
| **8**  Healthy mucosa | *HMGA2 / HPRT* | 1,59 | 0,06 | 0,05 |
|  | *Lin28 / HPRT* | 4.65 | 0.31 | 0.28 |
|  | *Let-7a / RNU6B* | 2.58 | 0.3 | 0.26 |
|  | *mir-98 / RNU6B* | 2.28 | 0.35 | 0.3 |
| **8**  Tumour | *HMGA2 / HPRT* | 12.4 | 1 | 0.8 |
|  | *Lin28 / HPRT* | 0.581 | 0.04 | 0.04 |
|  | *Let-7a / RNU6B* | 1.21 | 0.03 | 0.03 |
|  | *mir-98 / RNU6B* | 0.957 | 0.103 | 0.09 |
| **9**  Healthy mucosa | *HMGA2 / HPRT* | 1 | 0 | 0 |
|  | *Lin28 / HPRT* | 1 | 0 | 0 |
|  | *Let-7a / RNU6B* | 1 | 0 | 0 |
|  | *mir-98 / RNU6B* | 1 | 0 | 0 |
| **9**  Tumour | *HMGA2 / HPRT* | 5.92 | 0.37 | 0.35 |
|  | *Lin28 / HPRT* | 1.71 | 0.03 | 0.04 |
|  | *Let-7a / RNU6B* | 3.42 | 0.17 | 0.16 |
|  | *mir-98 / RNU6B* | 7.22 | 0.62 | 0.56 |
| **10**  Tumour | *HMGA2 / HPRT* | 9.39 | 1.41 | 1.27 |
|  | *Lin28 / HPRT* | 0 | 0 | 0 |
|  | *Let-7a / RNU6B* | 1.14 | 0.08 | 0.08 |
|  | *mir-98 / RNU6B* | 0.91 | 0.08 | 0.07 |
| **12**  Tumour derived Cell line | *HMGA2 / HPRT* | 55.1 | 1.9 | 1.8 |
|  | *Lin28 / HPRT* | 1.03 | 0.06 | 0.06 |
|  | *Let-7a / RNU6B* | 1.11 | 0.07 | 0.07 |
|  | *mir-98 / RNU6B* | 1.05 | 0.05 | 0.04 |
| **13**  Tumour derived Cell line | *HMGA2 / HPRT* | 44.5 | 1.4 | 1.3 |
|  | *Lin28 / HPRT* | 7.79 | 1.12 | 1 |
|  | *Let-7a / RNU6B* | 1.12 | 0.11 | 0.11 |
|  | *mir-98 / RNU6B* | 1.15 | 0.15 | 0.13 |
