# Supplementary material for: HMGA1 and HMGA2 expression and comparative analyses of HMGA2, Lin28 and let-7 miRNAs in oral squamous cell carcinoma
Source: BMC Cancer. 2014 Sep 23;14:694. doi: 10.1186/1471-2407-14-694 (PMC4190370; doi:10.1186/1471-2407-14-694)
Supplement: Supplementary file 4 — Additional file 4: Table S4: Comparative expression analyses of the HMGA2 and Lin28 genes and the let-7a and mir-98 miRNAs in canine OSCC Relative real-time PCR reactions were performed with canine HPRT and RNU6B as endogenous control genes. The non neoplastic palate sample obtained from patient 1 was used for calibration during data analyses. (DOC 60 KB) [file 12885_2013_4893_MOESM4_ESM.doc]

| **Canine Patient**  Sample | **Real-time PCR:**  Target gene / Endogenous control gene | **Expression**  **level** | **SD+** | **SD-** |
| --- | --- | --- | --- | --- |
| **1**  Healthy palate | *HMGA2 / HPRT* | 1 | 0 | 0 |
|  | *Lin28 / HPRT* | 1 | 0 | 0 |
|  | *Let-7a / RNU6B* | 1 | 0 | 0 |
|  | *mir-98 / RNU6B* | 1 | 0 | 0 |
| **2**  Healthy tongue | *HMGA2 / HPRT* | 1.19 | 0.24 | 0.21 |
|  | *Lin28 / HPRT* | 0.02 | 0.01 | 0.00 |
|  | *Let-7a / RNU6B* | 2.32 | 0.14 | 0.13 |
|  | *mir-98 / RNU6B* | 3.8 | 0.28 | 0.27 |
| **3**  Tumour | *HMGA2 / HPRT* | 42.9 | 1 | 1 |
|  | *Lin28 / HPRT* | 0.02 | 0.00 | 0.00 |
|  | *Let-7a / RNU6B* | 1.91 | 0.01 | 0.02 |
|  | *mir-98 / RNU6B* | 4.23 | 0.36 | 0.34 |
| **4**  Tumour | *HMGA2 / HPRT* | 7.52 | 0.43 | 0.41 |
|  | *Lin28 / HPRT* | 0.01 | 0.02 | 0.00 |
|  | *Let-7a / RNU6B* | 2.84 | 0.03 | 0.03 |
|  | *mir-98 / RNU6B* | 4.04 | 0.36 | 0.32 |
| **5**  Tumour | *HMGA2 / HPRT* | 5.86 | 0.83 | 0.72 |
|  | *Lin28 / HPRT* | 0.01 | 0.00 | 0.00 |
|  | *Let-7a / RNU6B* | 0.84 | 0.02 | 0.02 |
|  | *mir-98 / RNU6B* | 4.09 | 0.43 | 0.39 |
| **6**  Tumour | *HMGA2 / HPRT* | 3.09 | 0.16 | 0.16 |
|  | *Lin28 / HPRT* | 2.69 | 0.07 | 0.07 |
|  | *Let-7a / RNU6B* | 4.64 | 0.24 | 0.23 |
|  | *mir-98 / RNU6B* | 6.97 | 0.35 | 0.34 |
| **7**  Tumour | *HMGA2 / HPRT* | 24.7 | 1.2 | 1.2 |
|  | *Lin28 / HPRT* | 0 | 0 | 0 |
|  | *Let-7a / RNU6B* | 4.69 | 0.3 | 0.28 |
|  | *mir-98 / RNU6B* | 4.89 | 0.44 | 0.4 |
| **8**  Tumour | *HMGA2 / HPRT* | 11.1 | 0.5 | 0.4 |
|  | *Lin28 / HPRT* | 0.01 | 0.00 | 0.00 |
|  | *Let-7a / RNU6B* | 7.36 | 0.76 | 0.69 |
|  | *mir-98 / RNU6B* | 4.97 | 1.46 | 1.13 |
| **9**  Tumour | *HMGA2 / HPRT* | 178 | 10 | 11 |
|  | *Lin28 / HPRT* | 0.01 | 0.01 | 0.00 |
|  | *Let-7a / RNU6B* | 4.74 | 0.43 | 0.4 |
|  | *mir-98 / RNU6B* | 3.93 | 0.28 | 0.27 |
| **10**  Tumour derived Cell line | *HMGA2 / HPRT* | 242 | 2 | 3 |
|  | *Lin28 / HPRT* | 0.00 | 0.00 | 0.00 |
|  | *Let-7a / RNU6B* | 5.6 | 0.15 | 0.15 |
|  | *mir-98 / RNU6B* | 4.06 | 0.42 | 0.38 |
| **11**  Tumour derived Cell line | *HMGA2 / HPRT* | 270 | 8 | 7 |
|  | *Lin28 / HPRT* | 0.03 | 0.00 | 0.00 |
|  | *Let-7a / RNU6B* | 11.7 | 1 | 0.9 |
|  | *mir-98 / RNU6B* | 5.18 | 0.35 | 0.33 |
